# A backbone amide protecting group for overcoming difficult sequences and suppressing aspartimide formation

**DOI:** 10.1002/psc.2877

**Published:** 2016-04-18

**Authors:** Abu‐Baker M. Abdel‐Aal, George Papageorgiou, Richard Raz, Martin Quibell, Fabienne Burlina, John Offer

**Affiliations:** ^1^ The Francis Crick InstituteMill Hill LaboratoryThe RidgewayLondonNW7 1AAUK; ^2^ Sorbonne Universités, UPMC Université Paris 06, LBM4 place Jussieu75005ParisFrance; ^3^ CNRSUMR 7203, LBMParisFrance; ^4^ Département de Chimie, LBMENS, PSL Research University24 Rue Lhomond75005ParisFrance

**Keywords:** difficult sequence, aspartimide formation, *O*,*N* acyl transfer, backbone amide protection, Hmb, nitro reduction, reductive amination, Fmoc SPPS

## Abstract

A backbone amide bond protecting group, 2‐hydroxy‐4‐methoxy‐5‐nitrobenzyl (Hmnb), improved the synthesis of aggregation and aspartimide‐prone peptides. Introduction of Hmnb is automated and carried out during peptide assembly by addition of 4‐methoxy‐5‐nitrosalicylaldehyde to the peptidyl‐resin and on‐resin reduction to the secondary amine. Acylation of the hindered secondary amine is aided by the formation of an internal nitrophenol ester that undergoes a favourable *O*,*N* intramolecular acyl transfer. This activated ester participates in the coupling and generally gives complete reaction with standard coupling conditions. Hmnb is easily available in a single preparative step from commercially available material. Different methods for removing the amide protecting group were explored. The protecting group is labile to acidolysis, following reduction of the nitro group to the aniline. The two main uses of backbone protection of preventing aspartimide formation and of overcoming difficult sequences are demonstrated, first with the synthesis of a challenging aspartimide‐prone test sequence and then with the classic difficult sequence ACP (65‐74) and a 23‐mer homopolymer of polyalanine.

**Abbreviations**
Aa: amino acid
Boc: tert‐butyloxycarbonyl
CHCA: α‐cyano‐4‐hydroxycinnamic acid
DCM: dichloromethane
DIC: 1,3‐diisopropylcarbodiimide
DIEA: *N*,*N*‐diisopropylethylamine
Dmb: 2,4‐dimethoxybenzyl
DMF: *N*,*N*‐dimethylformamide
Fmoc: 9‐fluorenylmethyoxycarbonyl
HCTU: *O*‐(6‐Chlorobenzotriazol‐1‐yl)‐*N*,*N*,*N*′,*N*′‐tetramethyluronium hexafluorophosphate
Hmb: 2‐hydroxy‐4‐methoxybenzyl
HOBt: 1‐hydroxybenzotriazole
MALDI: matrix‐assisted light desorption ionization
MW: microwave
Pbf: 2,2,4,6,7‐pentamethyldihydrobenzofuran‐5‐sulfonyl
RP‐HPLC: reversed phase HPLC
r.t.: room temperature
SPPS: solid‐phase peptide synthesis
tBu: tert‐butyl
TFA: trifluoroacetic acid
TES: triethylsilane
Trt: trityl

## Introduction

**Figure 1:**
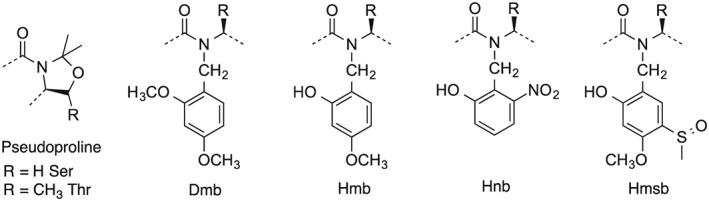
Backbone amide protecting groups discussed.

Backbone amide bond protection is the reversible alkylation of a peptide bond and the powerful solubilizing effect of this modification to peptides is well established. The improved synthesis of long, challenging peptides through the application of backbone protection has been the most convincing demonstration of its utility 1, 2, 3. The concept and beneficial properties of this substitution were originally introduced through the dimethoxybenzyl (Dmb) group (Figure 1) that masked the amide–amide hydrogen bonding potential for a peptide chain and thereby caused dramatically increased solubility of peptides 4, 5, 6. The same concept is now generally exploited to overcome the difficult sequences phenomena in solid phase peptide synthesis (SPPS) and to prevent aspartimide formation during Fmoc SPPS. This is achieved using a range of commercially available amide protecting groups, installed during the synthesis and removed during the final cleavage. Although adding another hydrophobic group to a peptide might be expected to decrease solubility, the major factor driving peptide insolubility is structurally based through formation of β − sheet architecture 7. Therefore, the simple masking of intermittent backbone amides prevents the formation of interchain hydrogen bonds, inhibits interchain aggregation and favours an open‐chain, disordered structure, with a concomitant improvement in solubility.

Attaching peptides to a solid phase is a general solution to improve their solubility. It solvates the protected peptides at concentrations they would not achieve unassisted in solution 8. The solid phase is not, however, a universal solution; poor solubility can manifest on solid phase as the difficult sequence problem. Difficult sequences are defined as a reproducible, sequence‐dependent collapse of the swollen resin volume accompanied by incomplete acylation and for Fmoc peptide synthesis, incomplete deprotection extending over several residues 9, 10. Although difficult sequences are also encountered with Boc synthesis they are addressed to a great extent by the *in situ* neutralization protocols developed by Kent and co‐workers 11. Difficult sequences can vary widely from mildly challenging to complete synthetic failure. In the extreme cases, such as alanine homooligopeptides, which exacerbate the phenomenon, the peptide becomes an insoluble aggregate attached to the resin incapable of further reaction 12. The cause of difficult sequences is interchain aggregation from amide–amide hydrogen bonding. Introduction of the tertiary amides of sarcosine and proline into polyalanine test sequences before the onset of aggregation significantly delayed aggregation 13. Inserting Dmb into the sequence also prevented aggregation. In addition, the protecting group could be removed in the cleavage step. However, this improvement was frustrated by another obstacle: the difficulty to acylate the sterically hindered secondary amine formed by the *N*‐substitution. A solution to this problem was intimated in the salicylimine models of the Kemp group 14, 15 and the salicylamide reactions of Brenner 16 that explored the efficient nature of intramolecular acyl transfer as a way to overcome hindered acylation. These concepts were subsequently incorporated into the design of the backbone protecting group, 2‐hydroxy‐4‐methoxybenzyl (Hmb) (Figure 1). The hydroxyl group of Hmb was first acylated through internally base catalyzed coupling followed by intramolecular acyl transfer to the secondary amine 17. However, although complete acylation was now observed for a wide range of amino acid pairings that formed the Hmb‐substituted amide bond, non‐standard coupling conditions of symmetric anhydride and dichloromethane (DCM) solvent were required with coupling times ranging from 1 to 48 h for complete acyl transfer 18, 19.

**Figure 2:**
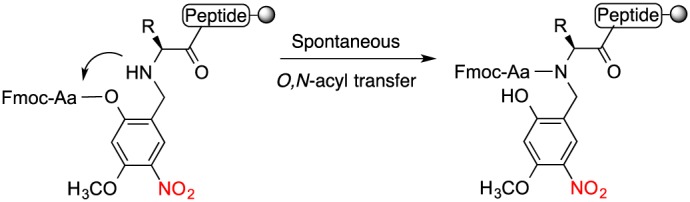
Backbone protecting group Hmnb, intramolecular *O*,*N* acyl transfer assisted by para nitro group.

The use of backbone protection became widespread with the availability of commercial dimethyloxazolidine derivatives of serine and threonine (pseudoprolines) 20, Dmb‐substituted dipeptide derivatives 2, 3 and Hmb‐substituted amino acid analogues (Figure 1). The former derivatives side‐stepped the need to acylate a sterically hindered secondary amine on the solid phase through their introduction as dipeptide building blocks 20. Virtually all the protected dipeptide combinations are now commercially available as pseudoprolines of Ser, Thr and protected pairings with DmbGly. The success of these reagents is due to their simple introduction using standard coupling conditions. However, they are restricted to substitution sites containing Ser, Thr and Gly (and in some cases Cys), and not all difficult sequences contain one of these residues at a convenient position before the onset of aggregation. In contrast, Hmb can theoretically be added at any position on the peptide backbone. Although complete acylation of Hmb substituted amino acids can be achieved, the use of Hmb‐substituted dipeptide derivatives was also explored to develop a more readily usable reagent akin to pseudoprolines and Dmb dipeptides. However, on activation, the phenolic hydroxyl of Hmb‐mono and dipeptide derivatives formed a poorly reactive 4,5‐dihydro‐8‐methoxy‐1,4‐benzoxapin‐2(3*H*)‐one precluding their use as dipeptide reagents 21. Ideally, a broadly applicable amide‐bond protecting group should be easily introduced with simple reagents, be rapidly acylated under typical reaction conditions and be easily removed as required. Observations from Hmb‐substituted amino acid and dipeptide studies suggested the possibility to improve the acyl‐transfer properties through introduction of an electron‐withdrawing group para to the 2‐hydroxyl of Hmb, and this was explored with Hmsb and related compounds (Figure 2). The resulting internal ester formed during coupling was now more activated and exhibited accelerated acyl transfer permitting the use of standard couplings. However, the Hmsb protecting group was now largely stable to trifluoroacetic acid (TFA) acidolysis. Reductive standard conversion of sulfoxide to sulfide restored acid lability 21. Many peptides have been prepared using Hmsb in an automated fashion 22, 23.

Accelerated acyl transfer has also been demonstrated for backbone protection using a nitro group as the electron‐withdrawing group. The nitro group is much more electron‐withdrawing than sulfoxide and therefore should show improved self‐acylation properties over Hmsb. It has been studied as 2‐hydroxy‐6‐nitrobenzyl 24 (Figure 1), which was photolabile and 2‐hydroxy‐4‐methoxy‐5‐nitrobenzyl (Hmnb) 21 (Figure 2), which was stable to photolysis and acid. We envisioned that the Hmnb group would become acid labile if the nitro group was converted (after acyl transfer) to an aniline group by reduction on the solid phase. The Hmnb precursor, 2‐hydroxy‐4‐methoxy‐5‐nitrobenzaldehyde, is available by a single, simple preparative step from 2‐hydroxy‐4‐methoxybenzaldehyde.

Our initial efforts focussed upon identification of a mild, peptide synthesis compatible, reductive method that would convert the aromatic nitro group to aniline and explore the acid lability of the group under different cleavage conditions. The use of a TFA/TMSBr/thioanisole mixture is attractive as it is a much stronger acid treatment than TFA alone but the mixture remains volatile and can be simply removed by sparging as for TFA 25.

## Materials and Methods

All reagents and solvents were purchased from Novabiochem® Merck‐Millipore or Sigma‐Aldrich. Fmoc‐Gly/Ala/Ile/Leu/Val‐OH, Fmoc‐Asp(OtBu)‐OH, Fmoc‐Gln‐OH, Fmoc‐Asn(Trt)‐OH, Fmoc‐Lys(Boc)‐OH, Fmoc‐Tyr(OtBu)‐OH and Boc‐Val‐OH were used. Peptides were prepared using DIC/HOBt (CEM Liberty Microwave Synthesizer) or HCTU/DIEA (CS Bio 336 automated synthesizer) activation for Fmoc/t‐Bu chemistry. Following peptide cleavage, the TFA‐cleavage cocktail was filtered; the filtrate sparged (N_2_) and peptides were precipitated with Et_2_O (Na‐dried, 4 °C). Peptides were purified by semi‐preparative HPLC on a RP‐C18 column (22 × 250 mm, Vydac) using linear gradients of CH_3_CN in 0.1% TFA/H_2_O with a flow rate of 5 ml min^−1^. HPLC gradients were prepared using solvent A as 0.1% TFA aqueous solution and solvent B as 90% CH_3_CN in 0.1% TFA aqueous solution. Detection was performed at 214 nm. Peptides were characterized by matrix‐assisted laser desorption ionization time‐of‐flight mass spectrometry (MALDI‐TOF MS) on a BRUKER microflex using CHCA matrix (10 mg ml^−1^ in CH_3_CN/H_2_O/TFA, 50 : 50 : 0.1 v/v). The ion positive reflector mode was used.

### 2‐Hydroxy‐4‐methoxy‐5‐nitrobenzaldehyde

2‐Hydroxy‐4‐methoxybenzaldehyde (24.2 g, 159 mmol) was dissolved in glacial acetic acid (48 ml) and heated on a water bath for 10 min. The solution was treated dropwise with concentrated HNO_3_ (8.6 ml, 191 mmol). When reaction ceased, the solution was cooled and a red precipitate formed. The solid was filtered, washed with cold acetic acid, and dried. Recrystallization from EtOH (charcoal) gave the desired product, 2‐hydroxy‐4‐methoxy‐5‐nitrobenzaldehyde (11.24 g, 36%), as pale fluffy needles, mp 172–173 °C. ^1^H NMR (400 MHz, CDCl_3_) δ, 11.75 (1H, s, OH), 9.78 (1H, s, CHO), 8.30 (1H, s, 6‐H), 6.56 (1H, s, 3‐H), 4.01 (3H, s, OCH_3_). The filtrate from the original reaction mixture was carefully diluted with water and a brown solid precipitated; this was filtered, washed with cold water and dried. Flash chromatography [EtOAc‐hexanes (2 : 3) to (1 : 1)] gave two products. First eluted product was a pale yellow solid (4.73 g). ^1^H NMR indicated that it was mostly unreacted starting material contaminated with some 3‐methoxy‐6‐nitrophenol. The second eluted product was *2‐hydroxy‐4‐methoxy‐3‐nitrobenzaldehyde* [1.35 g, 4% as light brown needles, mp 144–146 (EtOH), ^1^H NMR (400 MHz, CDCl_3_): δ 11.73 (1H, s, OH), 9.79 (1H, s, CHO), 7.65 (1H, d, *J* = 9.2 Hz, 6‐H), 6.67 (1H, d, *J* = 8.7 Hz, 5‐H), 3.98 (3H, s, OCH_3_)].

### *H*‐Val‐Lys‐Asp‐Gly‐Tyr‐Leu‐*NH_2_* (7)

The VKDGYL peptide was assembled on H‐Rink amide ChemMatrix® resin (Sigma‐Aldrich) (150 mg, resin loading 0.4–0.6 mmol/g) using DIC/HOBt activation (CEM Liberty1™ Single Channel Microwave Peptide Synthesizer). Fmoc deprotection was performed using 20% piperidine in DMF in two stages with an initial 0.5 min followed by a longer 3‐min treatment (7 ml, 40 W, 75 °C). Coupling reagents were as follows: Fmoc‐Aa‐OH/terminal Boc‐Val‐OH (0.2 M in DMF), DIC (0.8 M in DMSO) and HOBt (0.5 M in DMF). All coupling reactions were performed with fivefold excess Fmoc‐Aa‐OH/DIC/HOBt (1.0 : 0.8 : 2.0) and terminal Boc‐Val‐OH for 10 min, 25 W, 75 °C. Hmnb was installed onto Gly^4^: 2‐hydroxy‐4‐methoxy‐5‐nitrobenzaldehyde was added to the peptidyl resin (10 min, 1.1 eq, 0.02 M, 25 W, 50 °C) to form the imine followed by DMF flow wash. The imine was reduced with NaBH_4_ in DMF [2 × 15 min, 5 eq, 0.1 M, r.t.] followed by DMF flow wash. Asp^3^ was added using standard coupling. Synthesis yielded 230 mg of dried peptide‐resin. Test cleavage of the peptide‐resin **4** was performed using TFA/TES/H_2_O (95 : 2.5 : 2.5 v/v, rt. 1.5 h). Analytical HPLC gave a major peak as the target peptide with the Hmnb backbone protecting group characterized by MALDI‐TOF MS, *m*/*z* = 874.3 (first isotope), calc. *m*/*z* ([M + H]^+^) = 874.4. The MS profile showed a series of ions distinctive for nitro containing compound corresponding to the loss of one and two oxygens 26.

The peptide‐resin was swollen in DMF, filtered and mixed with freshly prepared solution of chromium(II) chloride in DMF (CrCl_2_, 0.04 M). Reaction conditions are reported in Table 1. The peptidyl‐resin was washed with DMF (3 × 5 ml), DCM (3 × 5 ml) and dried. The peptide‐resin was cleaved using a mixture of TFA/H_2_O/TES (100 : 5 : 2.5 v/v, 1 ml, 1.5 h). Analytical HPLC traces of the crude product gave a single peak as the target peptide with retention of the Ahmb group (**6a**) characterized by MALDI‐TOF MS, *m*/*z* = 866.0 (first isotope), calc. *m*/*z* ([M + Na]^+^) = 866.4. A portion of the peptide‐resin (25 mg) was cleaved with TFA/TMSBr/thioanisole/EDT (100 : 10 : 5 : 2.5 v/v, 1.5 h). Analytical HPLC traces of the crude product exhibit a single peak as the target peptide **7** without auxiliary characterized by MALDI‐TOF MS, *m*/*z* = 693.0 (first isotope), calc. *m*/*z* ([M + Na]^+^) = 693.0. HPLC conditions: RP‐C18, 5–35% CH_3_CN in 0.1% TFA over 30 min, 1 ml min^−1^. HPLC purification yielded 2.6 mg of peptide, 34.4% yield (purity > 98%).

**Table 1:** On‐resin reduction of nitro group of Hmnb

Entry	Nitro reduction method	% Nitro reduction^a^
1	Na_2_S_2_O_4_, K_2_CO_3_, TBAHS, DCM‐water (10 eq, r.t. 12 h)	5%
2	CrCl_2_, DMF (2x2.5 eq, r.t. 6 h)	100%
3	CrCl_2_, DMF (2.5 eq, r.t. 12 h)	75%
4	CrCl_2_, DMF (2.5 eq, r.t. 6 h)	60%
5	CrCl_2_, DMF (2.5 eq, r.t. 2 h)	30%
6	CrCl_2_, DMF (2.5 eq, 40 °C, 2 h)	65%
7	CrCl_2_, DMF (1.0 eq, 40 °C, 2 h)	8%
8	CrCl_2_, DMF (2.5 eq, 70 °C, 2 h)	100%
9	CrCl_2_, DMF (2.5 eq, 70 °C, 1 h)	100%

a Estimated from analytical HPLC.

Alternatively, the peptide‐resin (50 mg) was acetylated using acetic anhydride (0.16 mmol, 16 µl, 10 eq) and DIEA (0.08 mmol, 15 µl, 5 eq) in 300 µl DMF for 30 min. The solution was drained and the peptide‐resin treated with 20% piperidine/DMF, washed with DMF (3 × 5 ml), DCM (3 × 5 ml) and dried. Portions of the peptidyl‐resin were cleaved using a mixture of TFA/H_2_O/TES (100 : 5 : 2.5 v/v, 1 ml, 1.5 h) or TFA/TMSBr/thioanisole/EDT (100 : 10 : 5 : 2.5 v/v, 1 ml, 1.5 h). A summary of different cleavage conditions attempted on the peptide with Ac‐Ahmb or Ahmb is given in Table 2.

**Table 2:** Cleavage of backbone‐modified peptide

Peptide	Peptide cleavage	Ratio of 6a–b to 7
**5a**	TFA/TES/H_2_O	1 : 0
	TFA/TMSBr/thioanisole/EDT	0 : 1
**5b**	TFA/TES/H_2_O	3 : 1
	TFA/TMSBr/thioanisole/EDT	0 : 1

### *H*‐Val‐Gln‐Ala‐Ala‐Ile‐Asp‐Tyr‐Ile‐Asn‐Gly‐*OH*, ACP (65‐74), peptide 14

The ACP (65‐74) peptide was assembled on Fmoc‐Gly‐NovaSyn® TGT resin (Novabiochem®) (526 mg, resin loading 0.19 mmol/g) using HCTU/DIEA activation for Fmoc/t‐Bu chemistry (CS Bio 336 automated synthesizer). Fmoc‐deprotection was performed using 20% piperidine in DMF in two stages with an initial 3 min followed by 7‐min treatment. All coupling reactions were performed with fivefold excess Fmoc‐Aa‐OH/HCTU/DIEA (1.1 : 1.0 : 1.0) for 30 min. Hmnb was installed onto Ala^68^ by imine formation and reduction: 2‐Hydroxy‐4‐methoxy‐5‐nitrobenzaldehyde was added to the peptidyl‐resin to form the imine (30 min, 1.1 eq, 0.01 M) followed by DMF flow wash. The imine **9** was reduced with NaBH_4_ in DMF (15 min, ×5 eq, 0.1 M) to amine **10** followed by DMF flow wash. Ala^67^ was coupled directly to the Hmnb alkylated peptidyl‐resin under standard coupling conditions followed by DCM flow wash, 1 h shaking in DCM, DMF flow wash, 30 min re‐swelling in DMF. Gln^66^ was added as Fmoc‐Gln‐OH without trityl protection. On completion of the sequence the resin was washed with DCM and dried under vacuum yielding 565 mg of peptide‐resin **11**. Test cleavage of the Hmnb‐ACP peptide was performed using TFA/TES/H_2_O (95 : 2.5 : 2.5 v/v, rt. 1.5 h). Analytical HPLC gave a single peak as the target peptide with retention of Hmnb characterized by MALDI‐TOF MS, *m*/*z* = 1266.6 (first isotope), calc. *m*/*z* ([M + Na]^+^) = 1266.5.

The peptide‐resin (250 mg) was swollen in DMF, filtered and mixed with a freshly prepared solution of chromium(II) chloride (CrCl_2_, 0.04 M, 3.1 ml, 2.5 eq) at 70 °C for 2 h. The reaction mixture was drained, and the peptide resin was thoroughly washed with DMF (3 × 10 ml), DCM (3 × 10 ml) and dried.

The peptide‐resin (50 mg) was cleaved using a mixture of TFA/H_2_O/TES (100 : 5 : 2.5 v/v, 1 ml, 1.5 h). Analytical HPLC traces of the crude product showed a single peak as the target Ahmb‐ACP peptide **13** characterized by MALDI‐TOF MS, *m*/*z* = 1236.7 (first isotope), calc. *m*/*z* ([M + Na]^+^) = 1236.5. Alternatively, the peptide‐resin (50 mg) was cleaved with TFA/TMSBr/thioanisole/EDT (100 : 10 : 5 : 2.5 v/v, 1.5 h). Analytical HPLC showed a single peak as the target ACP (65‐74) peptide **14** characterized by MALDI‐TOF MS, *m*/*z* = 1085.6 (first isotope), calc. *m*/*z* ([M + Na]^+^) = 1086.5. HPLC purification yielded 3.2 mg of peptide, 21.3% yield (purity > 98%).

### Synthesis of *H*‐Ala_22_Val‐*OH*

The peptide was assembled on Fmoc‐Val‐NovaSyn® TGT resin (Novabiochem®) (170 mg, resin loading 0.19 mmol/g) using standard HCTU/DIEA activation for Fmoc/t‐Bu chemistry as described in the preceding texts (CS Bio 336 automated synthesizer). Backbone protection was inserted after Fmoc deprotection of Ala^7^, Ala^13^ and Ala^19^. Each backbone‐protecting group was introduced in two steps. 2‐Hydroxy‐4‐methoxy‐5‐nitrobenzaldehyde dissolved in DMF was added to peptide‐resin (1.1 eq, 0.01 M, 30 min) followed by DMF wash. Reduction with NaBH_4_ dissolved in DMF (filtered, PVDF 0.2 µm) (5 eq, 0.1 M, 15 min) was followed by thorough DMF wash. Synthesis yielded 190 mg of dried peptide‐resin. A portion of the peptide‐resin (50 mg) was cleaved using TFE/DCM (1 : 1 v/v, 1 ml, 1.5 h) and HPLC purification (30–50% B in 30 min) yielded 13 mg of peptide, (86% yield). The peptide was characterized by MALDI‐TOF MS, *m*/*z* = 2247.9, calc. *m*/*z* ([M + Na]^+^) = 2247.2.

## Results and Discussion

**Scheme 1:**
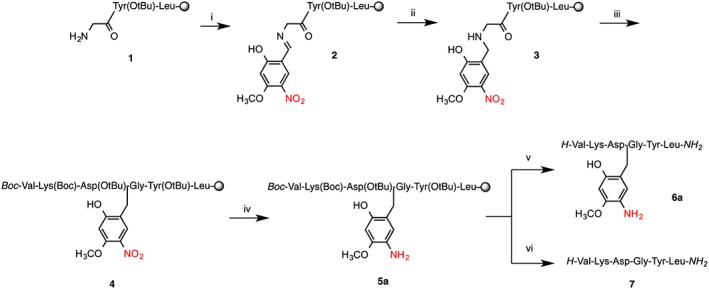
Incorporating Hmnb into synthesis of aspartimide‐prone sequence VKDGYL. (i) Imine formation; 2‐hydroxy‐4‐methoxy‐2‐nitrobenzaldehyde 1.1 equivalent to resin loading, followed by DMF wash; (ii) NaBH_4_/DMF; (iii) Fmoc‐Asp(OtBu)‐OH, DIC/HOBt 30 min, continue SPPS; (iv) Nitro reduction: CrCl_2_/DMF (2.5 eq, 70 °C, 2 h); (v) TFA/ TES/ H_2_O (95 : 2.5 : 2.5 v/v, rt. 1.5 h); (vi) TFA/ TMSBr/thioanisole/EDT (100 : 10 : 5 : 2.5 v/v, 1 h).

**Figure 3:**
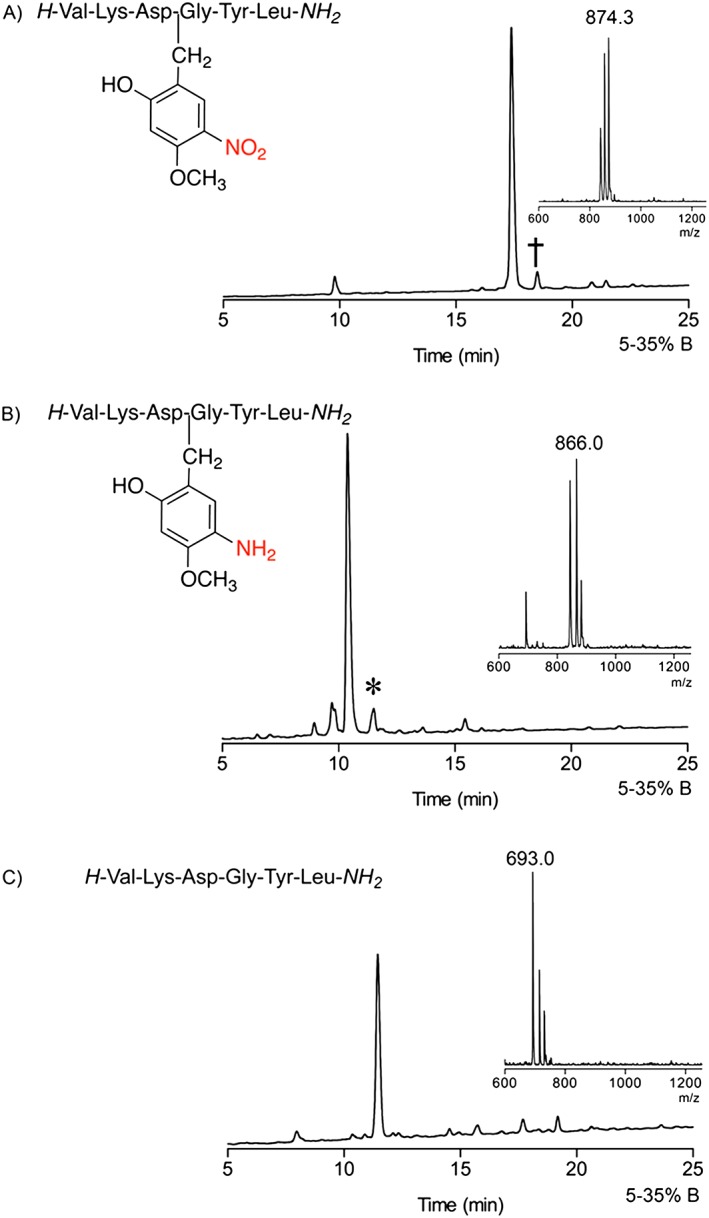
Analytical HPLC and MALDI‐TOF mass spectra of crude aspartimide‐prone peptide VKDGYL : (**A**) With Hmnb after cleavage of **4** with TFA/TES/H_2_O (95 : 2.5 : 2.5 v/v, rt. 1.5 h), calc. *m*/*z* = 874.4 for [M + H]^+^ ion (distinctive NO_2_ ion pattern 26) † non‐aspartimide side‐product, HPLC conditions: RP‐C18, 5–35 % CH_3_CN in 0.1% TFA over 30 min, 1 ml min^−1^. (B) Peptide **6a** with Ahmb after on‐resin nitro‐reduction with CrCl_2_ and cleavage of **5a** with TFA/TES/H_2_O (95 : 2.5 : 2.5 v/v, rt. 1.5 h), calc. *m*/*z* = 866.4 for [M + Na]^+^ ion. * peptide **7**. (C) Peptide VKDGYL **7** after cleavage of **5a** with TFA/TMSBr/thioanisole/EDT (100 : 10 : 5 : 2.5 v/v, 1.5 h), calc. *m*/*z* = 693.0 for [M + H]^+^ ion.

Aspartimide formation is the most serious side reaction of Fmoc SPPS, caused by repetitive piperidine treatments of Asp‐containing peptides. Prolonged and repeated base treatment of Asp‐Xaa sequences causes nucleophilic attack of the amide‐backbone onto the β‐carboxy of protected aspartic acid. The aspartimide can undergo nucleophilic ring opening caused by piperidine or water during subsequent steps producing further by‐products (L/D‐β‐aspartyl and D‐aspartyl peptides) that are difficult or impossible to separate from the product 27. Aspartimide formation is exacerbated by alternative bases such as DBU sometimes used for deprotection and by microwave synthesis. Backbone protection has been demonstrated to abolish aspartimide formation completely 28. The main contemporary approach is protection of the Asp‐Gly amide, by using commercially available derivatives such as the Fmoc‐Asp(OtBu)‐(Dmb)Gly‐OH building block 2. We have chosen a well‐established model for aspartimide formation derived from a scorpion toxin II peptide variant. Backbone protection is theoretically a universal solution to the aspartimide formation problem. It is also the only case where backbone protection is performing its eponymous role by suppressing reaction of the amide bond. Additionally, although the amide bond is generally considered stable, alkylation of the cysteine amide can favour *N*,*S* acyl transfer under ligation conditions and should be avoided 29. We have developed a generally applicable protocol based on Hmnb backbone protection to abrogate aspartimide formation (Scheme 1, Figure 3). The peptide was assembled on H‐Rink amide ChemMatrix® resin using DIC/HOBt activation for microwave‐assisted Fmoc/t‐Bu chemistry. The protocol is fully automated, and only three cycles were added to the standard SPPS to achieve backbone modification at Asp‐Gly; imine formation, imine reduction and post‐synthesis nitro reduction. All reactions are simple, quantitative, performed in DMF and compatible with standard SPPS. Imine formation was achieved with the addition of a slight excess of 2‐hydroxy‐4‐methoxy‐5‐nitrobenzaldehyde onto peptidyl‐resin **1**. Like most salicyaldehydes, 2‐hydroxy‐4‐methoxy‐5‐nitrobenzaldehyde forms exceptionally stable imines 22, 24, 30. Furthermore, the imine **2** was smoothly reduced to secondary amine using 5 equivalents of sodium borohydride. Imine formation is associated with a noticeable change of the resin colour to bright yellow, and imine reduction is accompanied by a loss of colour and evolution of hydrogen gas. All couplings were performed using standard SPPS reagents with single coupling of all amino acids to give the Hmnb substituted product **4**.

We observed that Hmnb backbone protection is stable to acid, even anhydrous HF. Test cleavage of the peptide‐resin **4** gave a crude product containing the Hmnb modified target peptide (Figure 3). A minor, later running side‐product was also observed with identical mass. We considered that reduction of the nitro after completion of the peptide synthesis to aniline would change the electronic properties of the benzyl group and could increase the acid lability of the backbone protection.

Several methods have been reported for peptide compatible solid‐phase reduction of nitroaromatic compounds including the use of sodium dithionite 31 and CrCl_2_ 32. The dithionite method requires the use of an aqueous solution, unsuitable for most resins. We tried different solvent mixtures with DMF, EtOH and dioxane at different ratios but no reduction was observed. We also tried using dithionite in a DCM/H_2_O solvent system with a phase transfer catalyst (Table 1, entry 1). At best, this gave 5% reduction. The CrCl_2_ reduction was simply performed by mixing the peptide resin with 2.5 equivalents of freshly prepared solution of CrCl_2_ in DMF overnight, followed by a thorough DMF wash. Reduction (60–75%) was observed, and reaction was complete after repeat treatment of the resin with CrCl_2_ (Table 1, entries 2–4). Hari and Miller used higher equivalents in their work 32. After optimization of reaction conditions (entries 5–9), 1 h reaction at 70 °C gave complete conversion (entry 9). This method should also be compatible with peptide synthesis performed on polyethylenglycol or polystyrene resins.

**Scheme 2:**
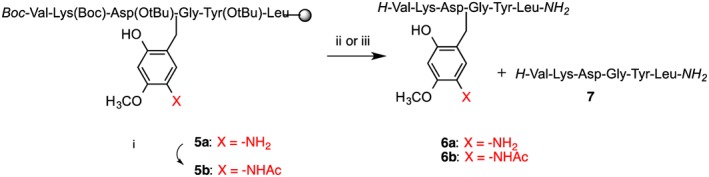
Ac‐Ahmb versus Ahmb (i) *N*‐acetylation: Ac_2_O (10 eq); DIEA (5 eq) in DMF for 30 min followed by 20% piperidine/DMF to remove acetylation of the Ac‐Ahmb hydroxyl. (ii) TFA/TES/H_2_O (95 : 2.5 : 2.5 v/v, rt. 1.5 h); (iii) TFA/TMSBr/thioanisole/EDT (100 : 10 : 5 : 2.5 v/v, 1 h).

Reduction of the nitro group of Hmnb opens the possibilities for further chemical modifications of the aniline. We wanted to explore the effect of aniline acetylation on the acid lability of the backbone protection during cleavage (Scheme 2). Peptide‐resins with Ahmb and Ac‐Ahmb (**5a–b**) were cleaved using two peptide cleavage methods: the standard TFA mixture and a TFA/TMSBr/thioanisole mixture (Table 2). The two protecting groups demonstrated different stability towards standard TFA mixture. For instance, TFA cleavage of **5a** gave predominantly the target peptide with Ahmb (a small amount of completely deprotected peptide **7** was also detected (Figure 3 **B**)) while **5b** gave a (3 : 1) mixture of the target peptide (with : without) the Ac‐Ahmb. In both cases, treatment of the peptide‐resin with the more acidic TFA/TMSBr mixture achieved complete removal of the auxiliary providing the target peptide **7** (Scheme 2, Table 2).

**Scheme 3:**
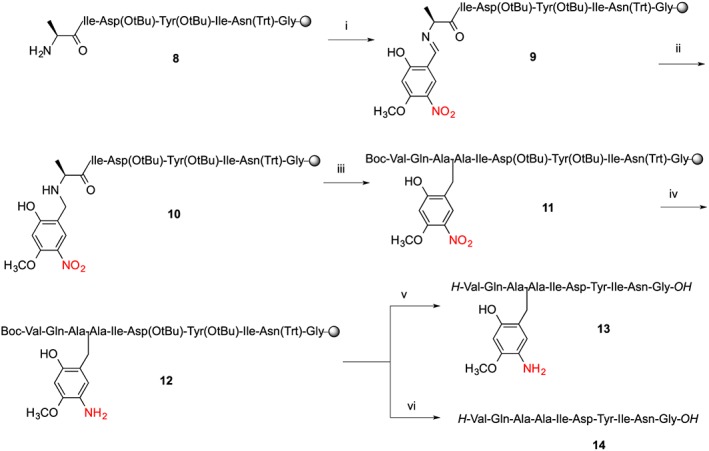
Automated synthesis of ACP (65‐74) using Hmnb backbone amide protecting group. (i) Imine formation; Hmnb 1.1 equivalent to resin loading followed by DMF wash; (ii) NaBH_4_/DMF; (iii) standard SPPS HCTU/DIEA coupling cycle, DCM 1 h, standard SPPS; (iv) nitro reduction: CrCl_2_/DMF (2.5 eq, 70 °C, 2 h); (v) TFA/TES/H_2_O (95 : 2.5 : 2.5 v/v, rt. 1.5 h); (vi) TFA/TMSBr/thioanisole/EDT (100 : 10 : 5 : 2.5 v/v, 1 h).

**Figure 4:**
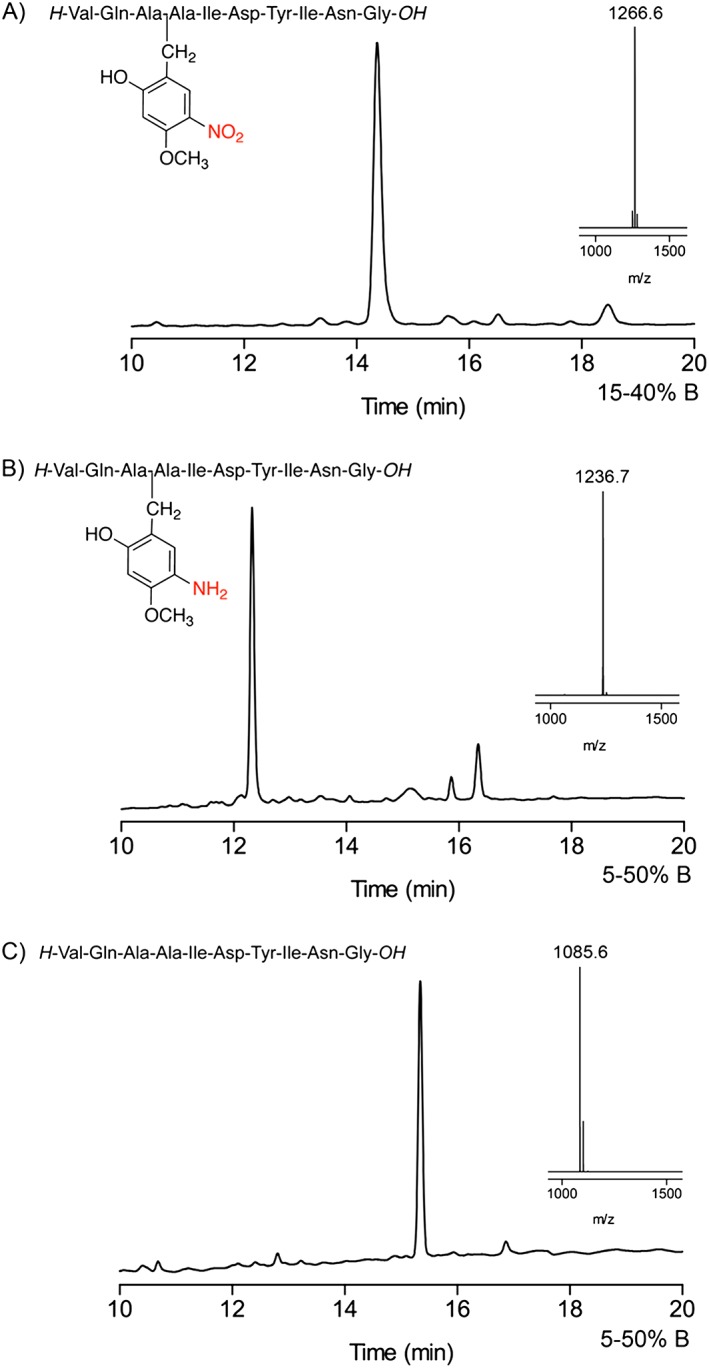
Analytical HPLC traces and MALDI‐TOF mass spectra of crude ACP (65‐74) peptide: (A) With retention of Hmnb after cleavage of **11** with TFA/TES/H_2_O (95 : 2.5 : 2.5 v/v, rt. 1.5 h), calc. *m*/*z* = 1266.5 for [M + Na]^+^ ion. (B) Peptide **13** with Ahmb auxiliary after on‐resin nitro‐reduction with CrCl_2_ and cleavage with TFA/TES/H_2_O (95 : 2.5 : 2.5 v/v, 0 °C, 1.5 h), calc. *m*/*z* = 1236.5 for [M + Na]^+^ ion. (C) ACP (65‐74) peptide **14** after cleavage of **12** with TFA/TMSBr/thioanisole/EDT (100 : 10 : 5 : 2.5 v/v, 1.5 h), calc. *m*/*z* = 1086.5 for [M + Na]^+^ ion.

ACP (65‐74) peptide is the classic difficult sequence and has been used extensively as a test peptide 23, 29, 33, 34, 35. Fmoc SPPS of ACP exhibits a characteristic delayed Fmoc deprotection and incomplete acylation with *N*‐terminal Val^65^. Introduction of Hmnb at Ala^68^, according to previous work with Hmb, should prevent peptide aggregation and therefore the des‐Val by‐product also. We were also interested to test the adaptability of the Hmnb protocols to a different resin type and coupling methods. The ACP (65‐74) peptide was assembled on Fmoc‐Gly‐NovaSyn® TGT resin using HCTU/DIEA activation (Scheme 3, Figure 4). Insertion of Hmnb (**8‐10**) was performed as previously described with a two‐step imine formation/reductive alkylation cycle onto Ala^68^. All couplings were carried out using standard SPPS – reagents at room temperature except for addition of Gln^66^ where Fmoc‐Gln‐OH was used with no trityl protection because the use of Fmoc‐Gln(Trt)‐OH alone largely prevents the onset of aggregation for ACP 13.

Test cleavage of **11** gave a crude product ACPHmnbAla^68^ lacking the Val deletion. On‐resin reduction of Hmnb‐ACP **11** to Ahmb‐ACP **12** was performed with 2.5 equivalents of CrCl_2_ in DMF at 70 °C for 2 h. Treatment of the Ahmb‐ACP peptidyl‐resin with TFA gave product **13** with Ahmb retained, while TFA/TMSBr/thioanisole treatment gave ACP(65‐74) **14** (Scheme 3, Figure 4). We are investigating more satisfactory alternatives to CrCl_2_ for reducing the nitro group to prevent the risk of metal contamination of the final product.

**Figure 5:**
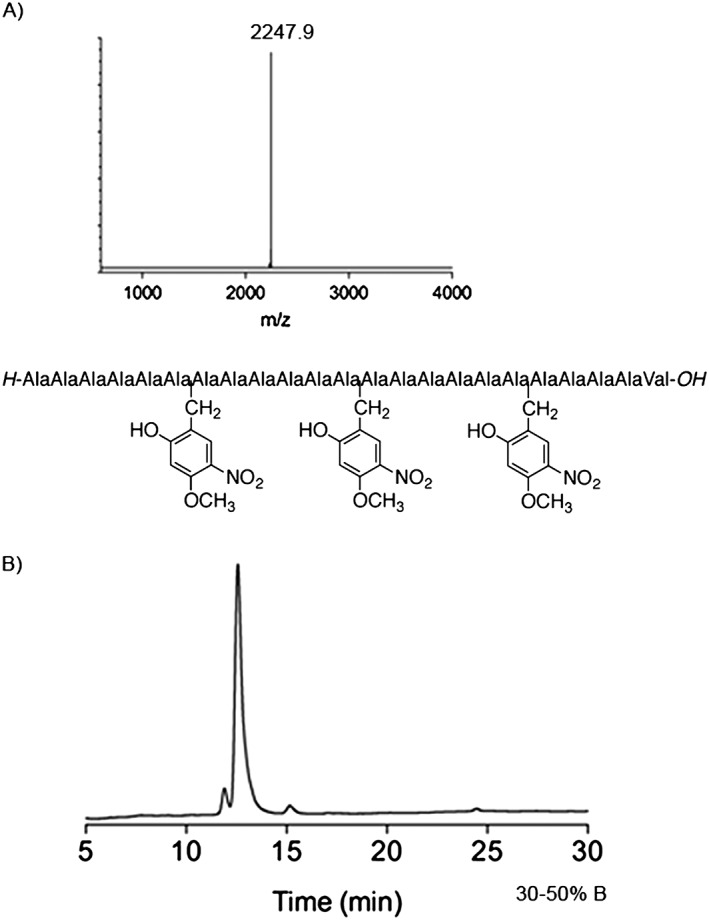
(A) MALDI‐TOF mass spectrum and (B) crude analytical HPLC trace of polyalanine 23‐mer peptide (*H*‐Ala_6_HmnbAla_6_HmnbAla_6_HmnbAla_4_Val‐*OH*) after cleavage with TFE/DCM (1 : 1 v/v), calc. *m*/*z* = 2247.2 for [M + Na]^+^ ion.

Polyalanine sequences are some of the most aggregation‐prone sequences known 12, 13. With polyalanine (*C*‐terminal Val) aggregation onset is early, starting after addition of the fifth residue. Hmnb was inserted at the fifth residue and at every sixth residue subsequently (Figure 5). The successful installation of three Hmnb groups by salicyldimine reductive amination cycles suggests that this reaction belongs to the exclusive set of solid phase applicable reactions. The handling and characterization of polyalanine are usually very difficult; however, with backbone protection a 23 mer of polyalanine has good solubility in standard HPLC solvents and can be easily characterized by MALDI‐TOF MS.

## Conclusion

We have developed a versatile backbone protection, Hmnb, which is easily available and can be introduced into a peptide synthesis in an automated fashion. Hmnb was installed on the peptidyl‐resin by an imine formation/reduction cycle. Both imine formation and reduction go to completion and give satisfactory incorporation on the solid phase. The nitro group assisted the acylation of the newly formed secondary amine by activating the intramolecular acyl transfer from the hydroxyl group to the secondary amine. Removal of Hmnb backbone protection was accomplished by conversion of Hmnb to the aniline analogue Ahmb on resin. Although both Hmnb and to a lesser extent Ahmb were stable to standard TFA treatment, Ahmb could be completely removed by cleavage with a mixture of TFA and TMSBr/thioanisole.
